# Cancer stem cells of hepatocellular carcinoma

**DOI:** 10.18632/oncotarget.24623

**Published:** 2018-05-01

**Authors:** Kewei Wang, Dianjun Sun

**Affiliations:** ^1^ Institute of Cell Biotechnology, China and Russia Medical Research Center, Harbin Medical University, Harbin, China; ^2^ Center for Endemic Disease Control, Chinese Center for Disease Control and Prevention, Harbin, China; ^3^ Key Laboratory of Etiology and Epidemiology (23618504), National Health and Family Planning Commission of the People’s Republic of China, Harbin, China; ^4^ Harbin Medical University, Harbin, China

**Keywords:** hepatocellular carcinoma, cancer stem cells, hepatic oval cells, apoptosis, CAR T-cells

## Abstract

Hepatocellular carcinoma is a malignant tumor arising from hepatocytes. The hepatocellular carcinoma is dictated by a subset of cells with stem cell-like features. These cells are apoptosis-resistant and have particular biomarkers, which serve as seeds in different stages of tumorigenesis including initiation, progression, metastasis, and relapse of hepatocellular carcinoma. Signaling pathways of cancer stem cells are novel targets for the radical intervention of hepatocellular carcinoma.

## BACKGROUND

**Figure 1:**
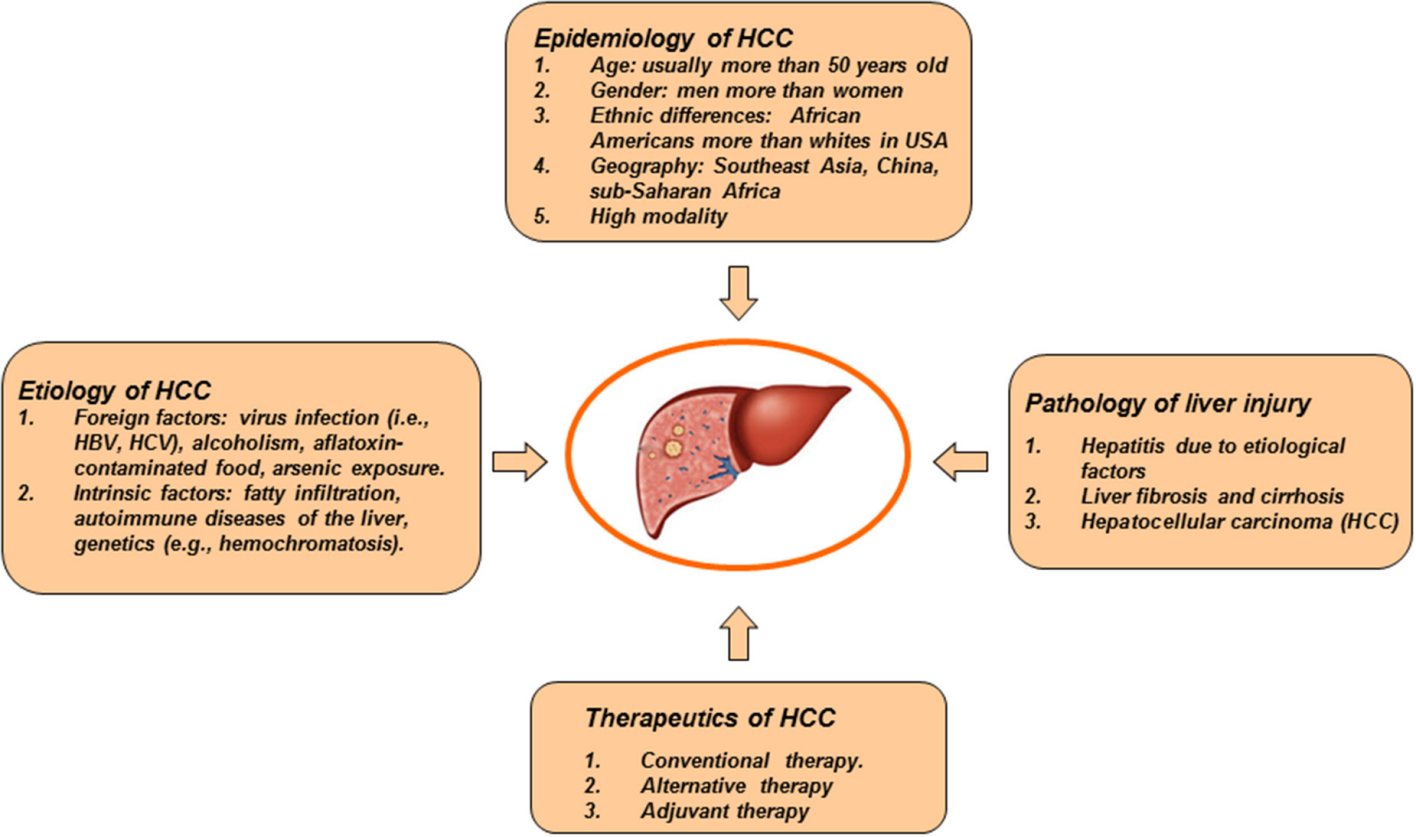
General characteristics of HCC HCC is a predominantly primary liver cancer. Etiology of HCC includes alcohol abuse, chronic infection of hepatitis B virus or hepatitis C virus, aflatoxin B1 intake, fatty infiltration, autoimmune diseases of the liver, arsenic exposure, hemochromatosis, and so on. In pathology, HCC has different growth patterns. Chronic hepatitis, cirrhosis and HCC are a typical pathological process of liver disease. HCC is one of the most aggressive diseases. Patients with advanced HCC have a poor prognosis.

Hepatocellular carcinoma (HCC) is a primary neoplasm that arises from hepatocytes. Approximately 90% of HCC cases are associated with known risk factors (Figure 1), such as alcohol abuse, chronic infection of hepatitis B virus (HBV) or hepatitis C virus (HCV), aflatoxin B1 intake, fatty infiltration, autoimmune diseases of the liver, arsenic exposure, and hemochromatosis [1]. These etiological factors may induce carcinogenesis via either independent or interactive mechanisms. Pathologists can distinguish several subtypes of HCC under microscopic observation. These subtypes seem to have different responses under treatment, but the prognosis of patients with advanced HCC remains unfavorable.

### Cancer stem cells of HCC

A small subset of tumor cells has the capacity to self-renew and differentiates into heterogeneous lineages of cancer cells, showing ‘stem cell-like’ characteristics. The concept on cancer stem cells was proposed 40 years ago. Technical advances in stem cell research have enabled to identify the minor cluster of tumor-initiating cells, which are termed cancer stem cells (CSCs). The first CSCs were found in acute myeloid leukemia [2]. Thereafter, the CSCs were continually recognized in a growing range of epithelial and other solid organ malignancies [3]. The definition of CSCs includes pluripotency that persistently gives rise to multiple cell types within cancer tissue [4]. Perhaps, a combination of diverse markers is needed to define a CSC population in tumor tissue. Properties of CSCs include persistent self-renewal, sustained proliferation, tumor initiation, rarity within tumor tissue, expression of stem cell markers, differentiation into multiple lineages, etc. Key CSC-specific genes that activate cellular hierarchy and stemness have been defined and some molecular signal pathways have also been detected in different tumors [5]. Some markers have been used to isolate CSCs that are responsible for relapse, metastasis, and chemoresistance of HCC. Different growth patterns of HCC may be determined by the contribution of CSCs. Some biomarkers of CSCs show unique features with phenotypes closely associated with committed liver lineages. Hepatic CSCs control a hierarchical network that participates in tumorigenesis. Distinct CSCs collaborate with the metastasis of HCC [6]. Altogether, liver CSCs have undergone genetic modification with excessive and persistent self-renewal. HCC arises from further proliferation and differentiation of liver CSCs.

### Potential origin of hepatic CSCs

#### CSCs from hepatocytes

**Figure 2:**
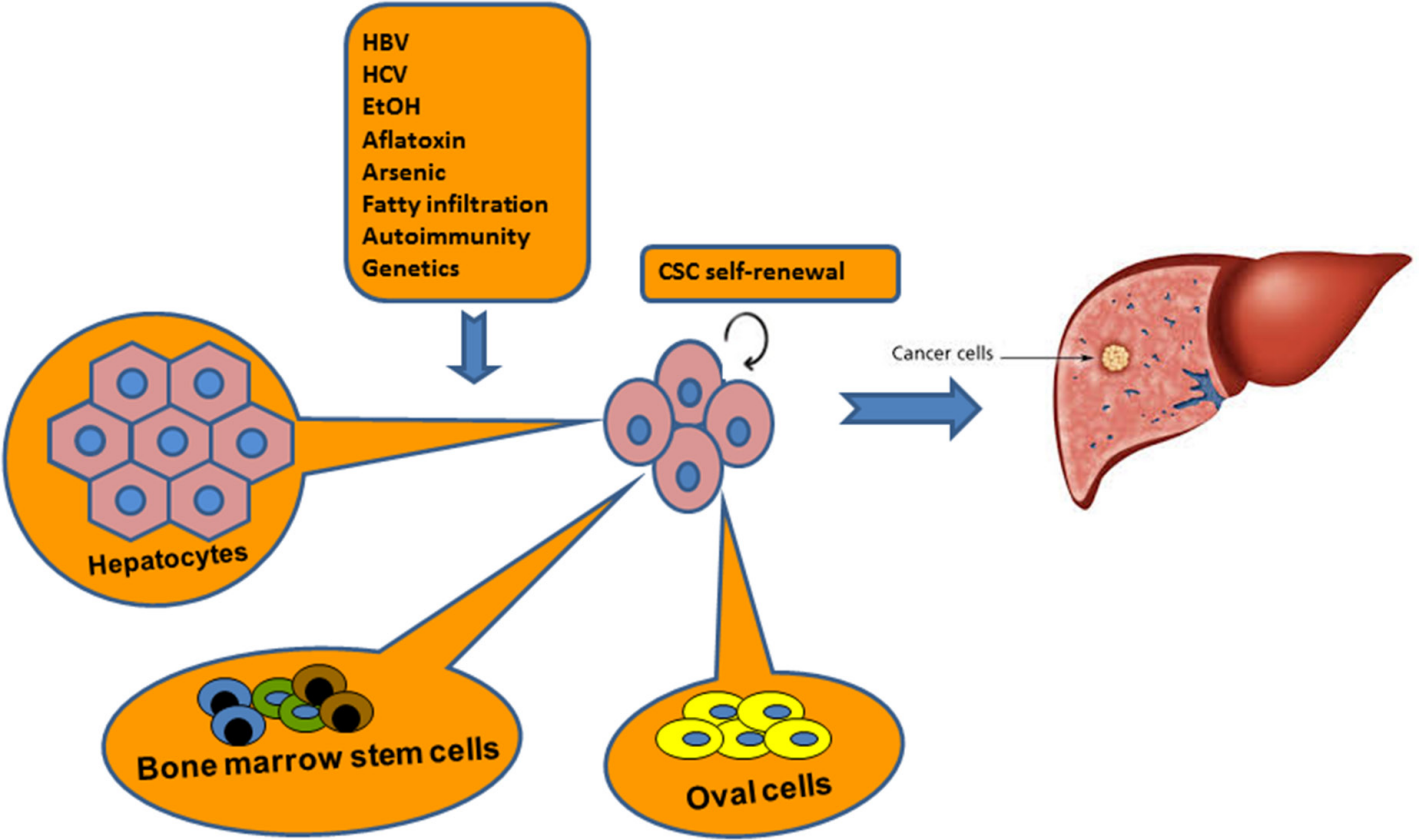
Origin of CSCs in pathogenesis of HCC It is believed that hepatic CSCs have undergone genetic modification with persistent self-renewal property. Hepatic CSCs may be originated from intrahepatic sources such as hepatocytes or oval cells, and extrahepatic bone marrow cells. However, no single cell type can perfectly explain the hypothesis of CSCs during development of HCC. Three cell types may be interacted each other to contribute the lineage of CSCs.

Experimental data demonstrated that hepatocytes were directly involved in hepatocarcinogenesis in 2-acetylaminflourene and diethylnitrosamine-treated rats via the labelled β-galactosidase [7, 8]. The mutated hepatocyte is possible source of cancer stem cells of HCC (Figure 2). Under physiological conditions, 0.01% of hepatocytes are in active cell cycle. Life expectancy of individual hepatocyte is over one-year period. When parenchymal cell loss occurs subsequent to partial hepatectomy, quiescent hepatocytes exit the G_0_ phase, enter the cell cycle, and express several preneoplastic markers, e.g. the placental form of glutathione-S-transferase and γ-glutamyl transferase [9]. Also, mature hepatocytes are the source of small hepatocyte-like progenitor cells (SHPCs) after Retrorsine treatment [10]. In liver injury owing to alcoholic or non-alcoholic fatty liver disease, appearance and activity of SHPCs are markers of disease severity [11]. The SHPC activation in chronic hepatitis correlates with the degree of inflammation [12]. There are 18.3% of regenerative hepatocytes originated from mature hepatocytes. Following a treatment of diethylnitrosamine, 17.7% of HCCs originated from mature hepatocytes. Above figures well match a hypothesis that cancer cells of HCC are from the proportion of mature hepatocyte-derived regenerative hepatocytes. This evidence demonstrates a direct lineage relationship between mature hepatocytes and HCC [9]. Presently, we need to know what factors induce gene mutation of hepatocytes and how it happens. Genetic factor or intrinsic risk (i.e., hereditary hemochromatosis and autoimmune hepatitis) definitely plays a critical role in pathogenesis of HCC. A recent research has revealed a strong correlation between tissue-specific cancer risk and the lifetime number of tissue-specific stem-cell divisions. This study demonstrates that intrinsic risk is better estimated by the low-bound risk controlling for total stem-cell divisions. Accumulated rates of endogenous gene mutation by intrinsic processes are not sufficient to account for the observed cancer risks. Intrinsic risk factors are thought to contribute only modestly (<10∼30% of lifetime risk) to cancer development. The remaining contribution (nearly 70∼90%) is provided by extrinsic risk factors. Thus, the extrinsic risk factors are substantial contributors to cancerogenesis [13]. For instance, chronic hepatitis can daily kill 0.3–3% of all hepatocytes [14]. The loss of hepatocytes parallels with the proliferative rate of hepatocytes, as evidenced by the 0.1–3.6% and 1–14% expression of proliferating cell nuclear antigen and antigen Ki-67 immunostaining respectively [15]. Actually, both HBV and HCV infection increase the risk of HCC by approximately 20-fold [16]. Patients with hepatitis B or C infection are at high risk of liver cancer. Nevertheless, the correlation between stem-cell division and cancer risk cannot distinguish the dominant effect of intrinsic factors from that of extrinsic factors. Currently, a definite interaction between intrinsic and extrinsic risk factors for the development of HCC remains to be determined. The hepatocyte undergoes an important transition called de-differentiation process by which the mature hepatocyte gives rise to the progenitor cells under the action of hepatocarcinogenic factors. HBV stimulates the production of CSCs-related markers (CD133, CD117 and CD90) and the expression of CSCs-related genes (Klf4, Sox2, Nanog, c-Myc and Oct4) in tumor cells [17]. Extrinsic factor HBx (hepatitis B virus X protein) contributes to the stem-like properties of OV6+ CSCs in HCC through the MDM2/CXCL12/CXCR4/beta-catenin signaling axis [18].

#### CSCs from hepatic oval cells (HOCs)

HOCs are a small subgroup of cells found in the liver. HOCs are characterized by ovoid nucleus and a high nucleus-to-cytoplasm ratio [19]. Generally, HOCs represent liver stress response or a secondary change when hepatic injury results in a decline of hepatocyte proliferation. Markers for the identification of oval cells include γ-glutamyl transpeptidase, glutathione-S-transferase, OV6, α-fetoprotein, neural cell adhesion molecule 1, and chromogranin A [20]. Interestingly, an activation of HOCs was correlated with the level of tissue damage and inflammation. The existence of HOCs has a great significance to the prognosis of clinical liver diseases [11]. HOCs can be induced and proliferated subsequent to a treatment of 2-acetylaminofluorene or hepatic injury (e.g. CCl_4_ administration or partial hepatectomy) [21]. Likewise, oval cells are activated in fatty liver disease of ob/ob mice or PARP1 knockout mice, in which the replication of mature hepatocytes is suppressed due to oxidative stress [22]. HOCs can also express a high level of Thy-1 that is not normally expressed in adult liver. Thy-1 is a cell surface marker used in conjunction with CD34 and lineage-specific marker to identify hematopoietic stem cells. Typical markers Thy-1, α-fetoprotein, γ-glutamyl transpeptidase, cytokeratin 19, and one cut homeobox 2, are all known for oval cell identification. Proliferation of HOCs could be stimulated by chemical carcinogen, 3’-methyl-4-dimethylaminoazobenzene in male rats. Using Thy-1 marker to isolate oval cells, a highly enriched population can be obtained [23]. The isolated HOCs were further identified via c-kit detection. By employing microarray hybridization, 22 miRNAs out of total 1,210 miRNAs were identified with a differential expression. The target genes of identified miRNAs were concentrated in angiogenesis, post-transcriptional protein modification, and small molecular metabolism. Differential expression of miRNAs demonstrates crucial roles of HOCs during the progression of HCC [24]. Another study also investigated pathological characteristics of HOCs and their relationship with the tumorigenesis of HCC in rats fed with 3, 3’-diaminobenzidine. Direct evidence that HOCs involved carcinogenesis of HCC was confirmed through a transplantation of the oval cells from p53-null mice into athymic nude mice [25]. Histologically, proliferated HOCs were initiated around the portal area as detected with c-kit and PCNA antibody. Then, oval cells gradually migrated into hepatic parenchyma. HOCs were distributed within entire tumor node and presented apparent features of undifferentiated cells, such as obvious nucleoli, high ratio of nucleolus-to-cytoplasm, and rare organelle in cytoplasm. Only some villus-like apophysis, few mitochondria or endoplasmic reticulum could be observed. The level of *c-myc* mRNA as a stimulator of HCC was increased with the progression of HCC. Evolvement of HOCs and *c-myc* expression may promote the development of HCC [26]. Anyway, no enough evidence confirms that hepatic oval cells are equal to cancer stem cells during tumorigenesis of HCC. Noticeably, oval cells express some markers of hematopoietic cells such as c-kit, flt-3, Thy-1 and CD34. Thus, it is speculated that the oval cells are derived from bone marrow precursor cells. However, such a speculation is questionable, considering oval cells are localized at the transitional zone between periportal hepatocytes and biliary cells of terminal bile ducts [19].

#### CSCs from bone marrow cells

Bone marrow is another potential source of HCC stem cells. Evidences include that (a) Y-chromosome-positive hepatocytes were identified in female acceptor liver subsequent to a transplantation of male bone marrow cells (BMCs). Y-chromosome specific probe was utilized *in situ* hybridization to confirm the presence of donor male BMCs. Moreover, a different degree of BMC engraftment was accompanied by a varied damage in parenchyma [27]. Clinical patients received gender-mismatched bone marrow or liver transplant. They showed significant frequencies of donor liver/bone marrow-derived cells [28]; (b) Granulocyte colony-stimulating factor could mobilize CD34+ stem cells into peripheral blood. These mobilized stem cells could further trans-differentiated into hepatocytes [29]; (c) BMCs contribute to the normal hepatocyte turnover process as proven by animal experiments. Oval cells/hepatocytes were occasionally derived from BMCs after liver damage using a lethally irradiated and bone marrow sex-mismatched transplant rat model [30]. When similar transplantation approach was utilized to trace the fate of BMCs in mice, it was found that about 1–2% of hepatocytes possibly derived from BMCs without liver injury [28]; (d) Hematopoietic BMCs can differentiate into functional hepatocytes expressing the enzyme fumaryl-acetoacetate hydrolase in tyrosinaemic (fah−/−) animals [29]. Moreover, a small number of lineage-negative BMCs with Sca-1+ (KTLS), c-kit+, and Thy-1-low was sufficiently able to generate functional hepatocytes in recipient animals [29]; (e) Damaged hepatocytes may alter the lineage commitment of hematopoietic stem cells towards hepatocytes, but only a low proportion of hematopoietic cells generate hepatocytes [31]. Another investigation discovered that functional hepatocytes were the result of the fusion between a donor bone marrow-derived macrophage and a fah−/− hepatocyte nucleus [32]. It should be noticed that genetically modified BMCs give rise to a low malignancy in chimeric mice [33]. Contradictorily, markers of BMCs were expressed only in lowly-differentiated cells (HA22T/VGH and SK-Hep-1). BMCs are an origin of poorly differentiated HCC. For instance, CD90 as a reliable marker of liver CSCs is shared by bone marrow-derived mesenchymal stem cells and by normal hepatic stem cells [34]. How does BMCs become hepatic CSCs? The mechanism remains unknown. Possible theories contain (a) genetic modification of BMSc under carcinogenic microenvironment; and (b) cell fusion is carried out between BMCs and liver CSCs. Actual role of BMCs in hepatocarcinogenesis remains controversial [35]. True significance of BMCs to HCC is far from being fully understood.

Collectively, hepatocytes, oval cells and BMCs may all be sources of liver CSCs. Each cell type is characterized by its peculiarity, but no single cell type can perfectly explain the hypothesis of CSCs in pathogenesis of HCC. All of above-mentioned cell types may be interacted each other to contribute the family of CSCs. The relevant investigation is still needed in future study.

### Markers of hepatic CSCs

**Figure 3:**
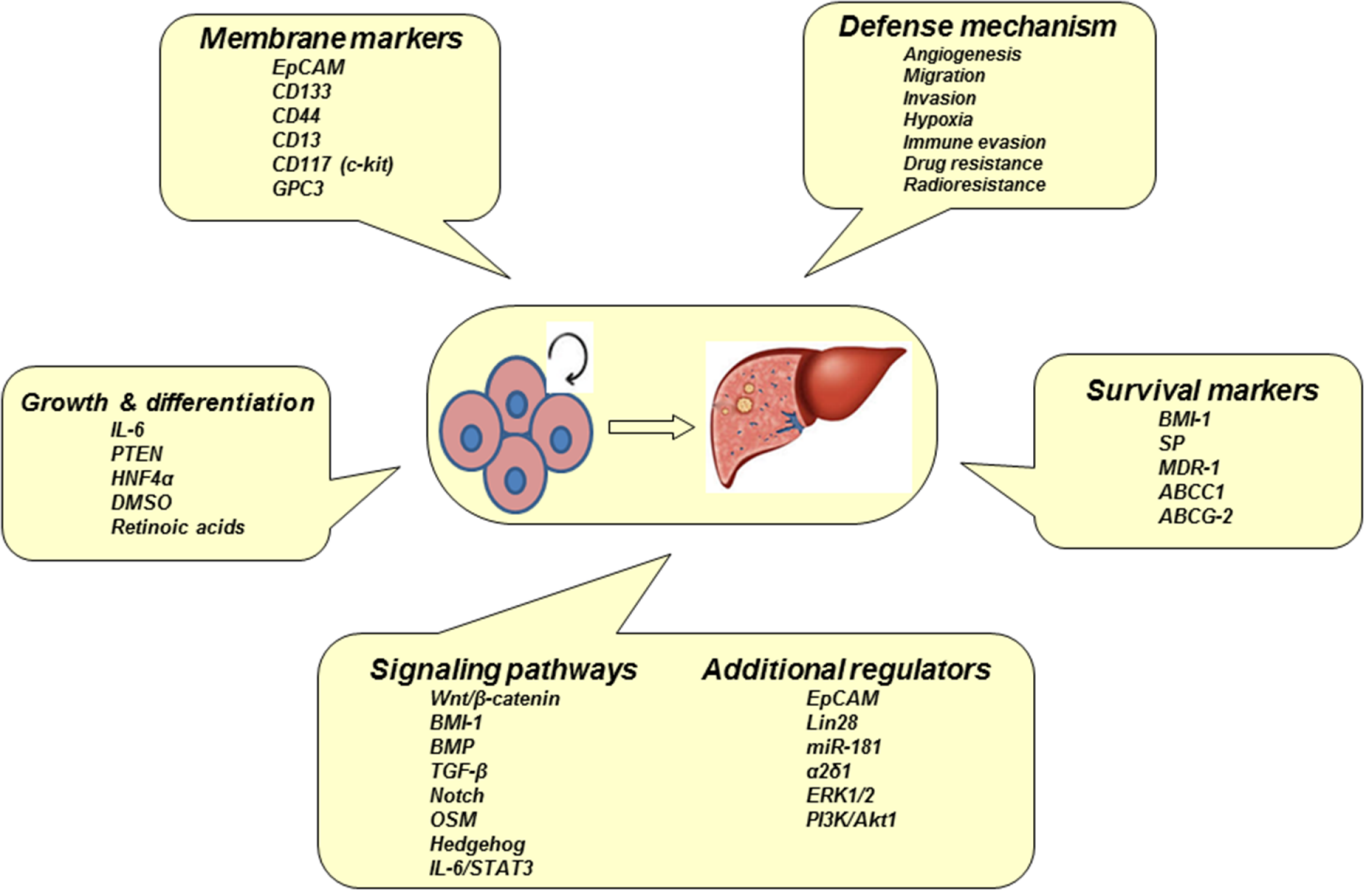
Biomarkers and signaling pathways of CSCs Hepatocarcinogenesis may be initiated from hepatic CSCs with a multistep process. Apoptosis-resistant CSCs in tumorsphere have particular biomarkers and their signaling pathways are activated by multiple regulators.

Hepatic CSCs defined by different markers show exclusive features of tumorigenicity and metastasis of HCC (Figure 3). These advances help us to understand the pathogenesis and heterogeneity of liver CSCs [6]. Previous study had indicated that tumor spheres were essentially enriched with CSCs. Actually, liver CSCs dictate a hierarchical network that is shared in both organogenesis and tumorigenesis [36]. Some surface markers for liver CSCs include CD133, CD105, CD90, CD45, CD44, CD13, and epithelial cell adhesion molecules (EpCAM). CD133(+) cells had a prominent ability to differentiate into heterogeneous lineages. CD133(+) cells also exhibited an increased potential for self-renewal as well as tumorigenesis [37]. CD133(+) subpopulation in HCC were more resistant to anticancer agents, such as doxorubicin and 5-fluorouracil [38]. Tumorsphere formed from HCC cells contained a high percentage of CD90(+) cells [39]. These CD90(+) cells show a high proliferation rate and low apoptosis rate as compared to control cells and were more tumorigenic and resistant to doxorubicin via the PI3K/Akt1 pathway [39]. Treatment with doxorubicin and PI3K/Akt inhibitors could increase apoptosis and reduced viability among cells in the tumorspheres. CD90 can be thus used as a potential biomarker for HCC CSCs. Moreover, activity of CD90(+) CSCs was enhanced through a stimulation of Notch pathway [40]. CD44(+) and CD133(+) were correlated with an enhanced AFP level as well as the risk of poorly differentiated HCC. CD44 or CD133 alone and in combination with microvascular invasion was independently associated with increased recurrence and poor prognosis of HCC patients [41]. The CD13 is another marker of CSCs. Ubenimex, a CD13 inhibitor, was combined with conventional anticancer drugs, fluorouracil, cisplatin, doxorubicin and sorafenib, which synergistically enhances their antitumor effects [42]. In addition, FGF19/FGFR4 is markers of hepatic CSCs within the fatty liver-steatosis-cirrhosis-HCC sequence. An overexpression of FGF19/FGFR4 is significantly correlated with EpCAM [43]. Upregulation of Notch2 was also discovered in CD90 positive HCC cells. The knockdown of Notch2 in HCC cells impaired the tumor formation *in vivo*. Notch2 is a potential therapeutic target for HCC [44]. GEP-expressing cells were isolated from clinical HCC samples, in which the cells exhibited higher levels of stem cell marker CD133, pluripotency-associated signaling molecules beta-catenin, Oct4, SOX2, Nanog, and chemodrug transporter ABCB5 [45]. Oct4 is a stem cell gene associated with hepatic CSCs, which increases the development of HCC [46]. So far, only a few markers (e.g., CD90, CK19, GPC3) of hepatic CSCs have been well understood [34, 47, 48]. Ongoing investigation focuses on various regulators of CSC phenotype, which are classified into (a) small RNA molecules. Upregulation of miR-500a-3p promotes the persistent maintenance of CSC phenotype via targeting multiple negative regulators (i.e., SOCS2, SOCS4, PTPN11) of JAK/STAT3 signaling pathway [49]. A highly expressed lncRNA (ICAM-1-related) specifically regulates CSC properties of ICAM-1(+) HCC cells [50]; (b) transmembrane protein. It is known that expression of CLAUDIN-1 can abrogate CSC-Like behaviors in HCC [51]; (c) transcription factor. The protein SRY (sex determining region Y)-box 9 (Sox9) is required for tumor initiation and cell division in liver CSCs [52]; and (d) other potential biomarkers. Dead end 1 (Dnd1) can activate Hippo pathway in HCC cells to inhibit CSC-like characteristics [53]. Tg737 gene is able to inhibit CSCs invasion and migration of HCC in an ERK1/2/MMP-2 dependent manner [54]. Mammalian-enabled (MENA) protein as an actin-regulatory protein is associated with high mRNA levels of CD133, CK19, and EpCAM in human HCC tissues. [55]. At present, no single marker is enough to define the phenotype of CSCs. Further investigation for particular markers and signal pathways is still necessary to identify the property of CSCs using established HCC cell lines and animal models.

### Signaling pathways of hepatic CSCs

The process of hepatocarcinogenesis is multi-causal and multi-factorial. Primary mediators of HCC consist of HBV or HCV infection, alcohol, and metabolic liver diseases, and so forth. HCC as one of solid tumors is composed of a heterogeneous population of cells. The CSCs may result from genetic and epigenetic modification of hepatocytes, hepatic oval cells, or circulating bone marrow cells. These modified cells together with a deregulation of the microenvironment (e.g., chronic inflammation) result in a distinct lineage of CSCs that have stem-like features. Although the existence of CSCs can be identified by their biomarker, the mechanism for CSCs to work on the development of HCC remains unknown. How do liver CSCs trigger cancer initiation? Hepatocarcinogenesis as a multistep process is started by liver injury (due to chronic hepatitis and concomitant cirrhosis) and then liver regeneration. The regeneration subsequent to liver injury induces an activation of normal liver stem/progenitor cells [56]. An excessive and persistent self-renewal of signal is one of key events in the early stage of carcinogenesis [57]. Deregulation of the self-renewal signal in liver stem cells is a potential mechanism in hepatocarcinogenesis. Experimentally, two major self-renewal regulators Wnt/β-catenin and BMI-1 were activated to drive tumor formation using purified liver stem/progenitor (c-kit−CD49+CD45−Ter119−) cells [58]. Wnt/β-catenin, BMI-1, TGF-β, Notch and Hedgehog signaling pathways not only are stem cell activators to expedite tumorigenesis, but serve as molecular targets to fight against cancer as well. Additional factors such as EpCAM, Lin28 or miR-181, target CSCs to contribute to progression of HCC. The CSCs of HCC also overexpress α2δ1 subunit of calcium channel, and thus α2δ1 or its downstream mediators (e.g., ERK1/2) may be targets to kill CSCs [59]. The maintenance of CSCs also needs other factors such as angiogenesis, invasion, hypoxia, immune evasion, drug resistance, and radioresistance. All of above-mentioned factors can be considered for designing therapeutic schemes of HCC. CSCs have a complicated signaling network with a considerable crosstalk and redundancy. Thus, targeting single molecule or pathway has only a limited benefit for treatment. Moreover, some signal molecules or pathways are shared by both CSCs and normal stem cells, which increase the difficulty in choosing targets to aim at CSCs but spare normal stem cells. Actually, CSCs can survive conventional therapy, resulting in HCC recurrence and tumor relapse.

### Novel therapeutics based on CSC targets

**Figure 4:**
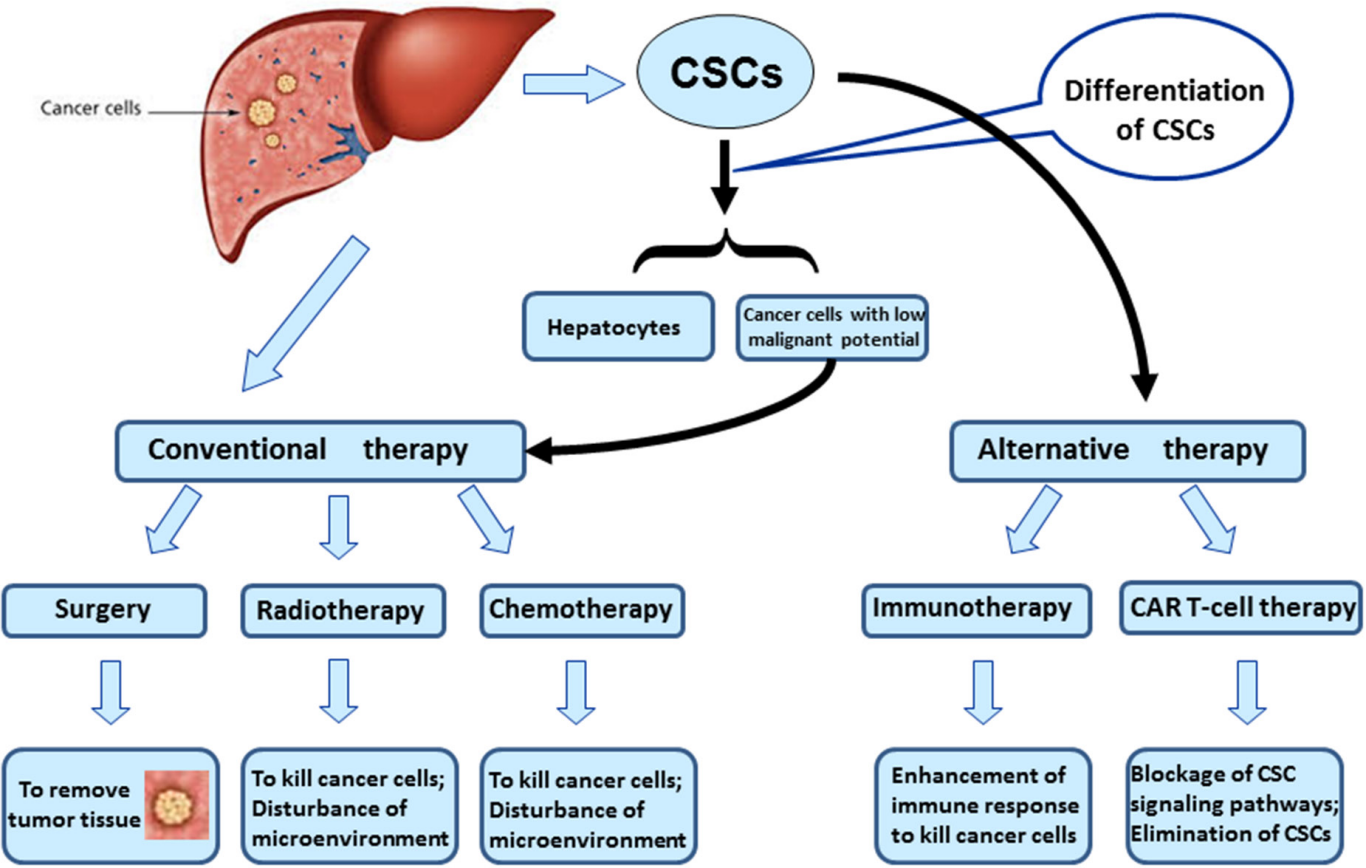
Novel therapeutics based on CSC targets Strategies include (i) CAR T-cell technology to eliminate CSCs directly. (ii) To promote differentiation of hepatic CSCs into cancer cells with low-malignant potential. Those differentiated cancer cells are sensitive to conventional antineoplastic therapy.

In recent years, targeting intervention on liver CSCs has been becoming a novel strategy for HCC treatment (Figure 4) [60]. In a rat model of liver carcinogenesis, CD133(+)CD44(+)CD45(-)HIS49(-) cells were a fraction that was able to be converted into hepatic oval cells. An acyclic retinoid that improved overall survival after HCC tumor removal, could directly inhibit the extensive expansion of the isolated precancerous cells *in vitro* and decreased the emergence of the precancerous cells and their progeny *in vivo*. Long-term observation confirmed the reduction in precancerous changes after the acyclic retinoid treatment, ultimately resulting in suppression of HCC development. The CD133+CD44+ precancerous subpopulation of oval cells is a therapeutic target for HCC [61]. Therefore, CSCs-directed therapeutic approaches may represent strategies to improve clinical treatment of HCC. It was found that BC047440 played a critical role in mediation of HCC cell proliferation. BC047440 expression was markedly upregulated in liver CSCs. An inhibition of BC047440 could stimulate cell proliferation through activation of NF-κB and the level of HNF4α was increased. Importantly, an inhibition of BC047440 could promote the differentiation of liver CSCs into hepatocytes. Tumorigenicity suppressed subsequent to BC047440 depletion was also confirmed in the nude mouse model. The knockdown of BC047440, resulting in proliferation inhibition and differentiation induction of hepatic CSCs, is a novel therapeutic target of HCC [62]. Chimeric antigen receptor (CAR) T cells are the engineered lymphocytes that express specific receptors. CAR T-cells have cancer-killing ability as bound to particular marker proteins of CSCs, such as CD19, CD20, and CD22 [63]. The “programmed” CAR T-cells are expanded in the body for a long time and act like “living drug”. Thus, the CAR T-cell technology has a great potential in the treatment of cancer. Glypican-3 (GPC3) as tumor antigen was exploited to develop GPC3-targeted CAR-T cells for the treatment of HCC. The third generation of GPC3-targeted CAR T-cells was prepared by employing lentiviral transduction, which could efficiently kill GPC3-positive cancer cells *in vitro* and eradicated HCC xenografts expressing high level of GPC3 *in vivo*. The survival rate of mice was significantly enhanced subsequent to an administration of GPC3-targeted CAR T-cells. The GPC3-targeted CAR T-cell technology is a potential tool for therapeutic intervention of GPC3-positive HCC. Another strategy is to differentiate hepatic CSCs into new status with low-malignant potential, which is sensitive to conventional therapy. Moreover, approaches to disrupt stem cell niche and microenvironment essential for CSC homeostasis are also thought of as additional strategy for cancer treatment. The relative investigation leading to effective therapy of HCC is under way.

## CONCLUSION

The development of malignant HCC is dictated by a subset of CSCs. Hepatic CSCs have particular biomarkers combined in signaling network, which may be novel targets for the radical intervention of HCC.

## Abbreviations

HCC: hepatocellular carcinoma
HOCs: hepatic oval cells
BMCs: bone marrow cells
TUNEL: terminal deoxynucleotidyl transferase dUTP nick-end labeling
HBV: hepatitis B virus
HCV: hepatitis C virus
CSCs: cancer stem cells
AFP: alpha-fetoprotein
GEP: granulin-epithelin precursor
CCl_4_: carbon tetrachloride
PCNA: proliferating cell nuclear antigen
Thy-1: thymocyte differentiation antigen 1
CK19: cytokeratin 19
GPC3: Glypican 3
NF-κB: nuclear factor kappa-light-chain-enhancer of activated B cells
HNF4α: hepatocyte nuclear factor 4α
CAR T-cells: chimeric antigen receptor T cells
